# Pressure Induced Densification and Compression in a Reprocessed Borosilicate Glass

**DOI:** 10.3390/ma11010114

**Published:** 2018-01-12

**Authors:** Kathryn J. Ham, Yoshio Kono, Parimal J. Patel, Steven M. Kilczewski, Yogesh K. Vohra

**Affiliations:** 1 Department of Physics, University of Alabama at Birmingham, Birmingham, AL 35294, USA; katieham@uab.edu; 2 High Pressure Collaborative Access Team, Geophysical Laboratory, Carnegie Institution of Washington, Argonne, IL 60439, USA; ykono@uab.edu; 3 Ceramics and Transparent Materials Branch, U.S. Army Research Laboratory, Aberdeen Proving Ground, Aberdeen, MD 21005, USA; parimal.j.patel.civ@mail.mil (P.J.P.); steven.m.kilczewski.ctr@mail.mil (S.M.K.)

**Keywords:** borosilicate glasses, high-pressure, equation of state of material, X-ray radiography, X-ray diffraction

## Abstract

Pressure induced densification and compression of a reprocessed sample of borosilicate glass has been studied by X-ray radiography and energy dispersive X-ray diffraction using a Paris-Edinburgh (PE) press at a synchrotron X-ray source. The reprocessing of a commercial borosilicate glass was carried out by cyclical melting and cooling. Gold foil pressure markers were used to obtain the sample pressure by X-ray diffraction using its known equation of state, while X-ray radiography provided a direct measure of the sample volume at high pressure. The X-ray radiography method for volume measurements at high pressures was validated for a known sample of pure α-Iron to 6.3 GPa. A sample of reprocessed borosilicate glass was compressed to 11.4 GPa using the PE cell, and the flotation density of pressure recovered sample was measured to be 2.755 gm/cc, showing an increase in density of 24%, as compared to the starting sample. The initial compression of the reprocessed borosilicate glass measured by X-ray radiography resulted in a bulk modulus of 30.3 GPa in good agreement with the 32.9 GPa value derived from the known elastic constants. This method can be applied to variety of amorphous materials under high pressures.

## 1. Introduction

The materials for transparent armor applications are generally silicate based either in the form of glass or glass ceramics, and can exist in many different crystalline and amorphous modifications. The ballistic impact response of transparent armor materials requires an understanding of densification process under applied stresses, yield stress, and failure mechanisms under dynamic shock loading. A direct measurement of densification and compression produced under high pressure provides critical data for modeling ballistic response of transparent armor materials and in design of novel armor materials. The equation of state of a material provides information about the thermodynamic state of a system under specified physical conditions [1]. For crystalline materials with a well defined unit cell and long range order, the volume and pressure can be determined from X-ray diffraction experiments and a known equation of state of a pressure standard, respectively [1,2,3]. Since amorphous materials lack long range order, X-ray diffraction cannot be used as a method to obtain direct sample volume measurements. Attempts have been made to directly measure the equation of state of solids under diamond anvil cell (DAC) compression using optical microscopy [3]. The main drawback of this method is the use of irregularly shaped samples, which are on the order of tens of microns in diameter. Another technique, X-ray microtomography, has been used to determine sample densification by rotating the sample in 0.125° increments to obtain a sequence of three-dimensional (3D) tomographic images; this method has the drawback of being more time intensive, as the sample is rotated through multiple angles [4]. By comparison, X-ray radiography method [5,6] has been developed for bulk millimeter size samples using Paris-Edinburgh (PE) press at Beamline 16-BM-B (HPCAT) at the Advanced Photon Source, Argonne National Laboratory. In our research, we have adapted this PE press for carrying out X-ray radiography on reprocessed borosilicate glasses, with images of 1936 px × 1216 px with resolution of $\frac{0.850\;µm}{1\;pixel}$. The radiography technique used in this work has the advantage of much shorter experiment times, as a cylindrical sample geometry is used, which leads to a volume calculation from the sample height and width from just 1 radiograph per pressure step, which can be obtained in under a second. Radiography studies of samples at Beamline 16-BM-B can also be done in conjunction with energy-dispersive X-ray diffraction, allowing for pressure determination from measured volume of the gold pressure standard. The X-ray radiography and multi-angle energy dispersive X-ray diffraction technique has been recently applied to a high-boron content borosilicate glass; however, direct sample volume measurements were not possible in this study due to limitations of the sample assembly [7]. 

## 2. Results

**Figure 1:**
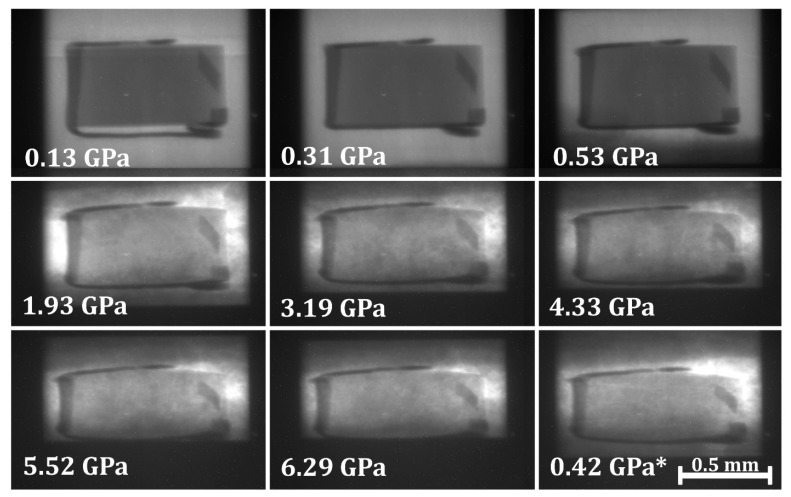
White-beam X-ray radiography images of α-Fe at increasing pressures and on decompression (noted with an ‘*’). The gold foil represented by the dark features represents the vertical and horizontal extent of the α-Fe sample.

For validation of experimental technique, white-beam X-ray radiography was conducted on a cylindrical sample of α-Fe (stable in the body-centered cubic phase) from ambient condition to a maximum pressure of 6.3 GPa. Radiography images were taken at increasing pressure steps, as seen in Figure 1. The sample was decompressed from 6.3 GPa to 0.4 GPa, and the decompression radiography image is denoted by an ‘*’ in Figure 1.

**Figure 2:**
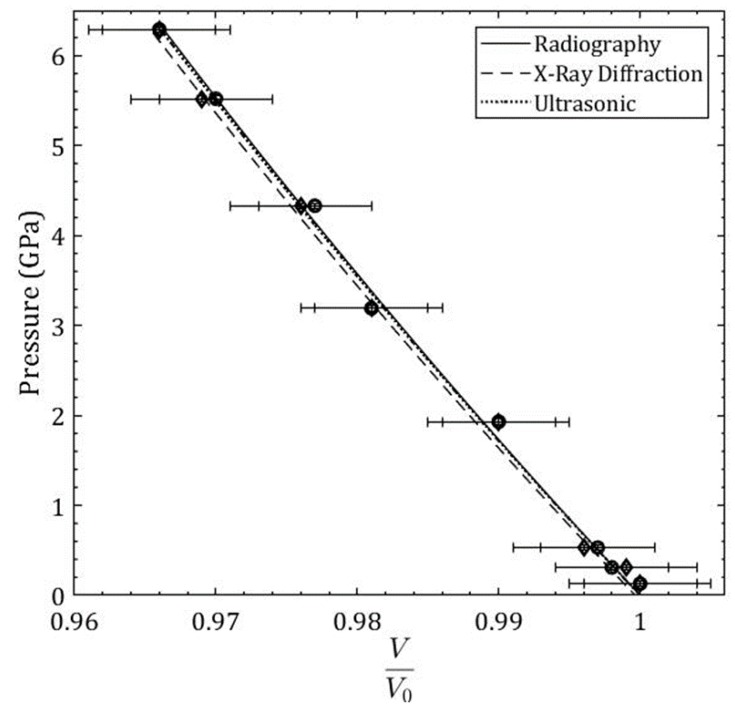
A comparison of Pressure-Volume (P-V) data or equation of state of α-Iron obtained by X-ray radiography and X-ray diffraction methods. The equation of state derived from ultrasonic data is also shown. The experimental data points obtained with the X-ray diffraction experiment are (♦) and the experimental data points obtained with the radiography experiment are (●).

The radiography images were used to obtain the length and width values at each incremental pressure step. The sample height *H* and sample width *W* at each pressure step were measured from the top gold foil to the bottom gold foil and from the left gold foil to the right gold foil, respectively (in pixels). These measurements were then converted to millimeters using the conversion factor $\frac{0.850\;\mu m}{1\;\mathrm{pixel}}$. The initial ambient image at *p* = 0.13 GPa (Figure 2) was taken when the top PE anvil is closed onto the sample assembly, but oil pressure has not been applied yet. The heights and widths were normalized by the initial height $H_{0}$ and $W_{0}$, respectively. $\frac{H}{H_{0}}$ decreases with an increasing pressure, while $\frac{W}{W_{0}}$ increases with increasing pressure. The volumes were calculated as $V=\pi {\left(\frac{W}{2}\right)}^{2}H$ with the raw height and width values in pixels, then normalized by the initial volume $V_{0}$, such that $\frac{V}{V_{0}}$ = 1 (shown in Figure 2). 

Gold foil (2 µm thick, shown in Figure 1 as the dark outline around the α-Fe sample) was used as the pressure standard and the marker in the white-beam X-ray radiography direct volume measurement. Multiple energy-dispersive X-ray Diffraction (EDXD) spectra were collected for each incremental pressure step from the top, bottom, and side gold foil markers and then averaged to determine the sample pressure. The third-order Birch-Murnaghan equation of state [1] was used to determine the bulk modulus $B_{0}$ and the first derivative of the bulk modulus $B_{0}^{\prime }$ from experimental white-beam X-ray radiography experiments of pure α-Fe. A nonlinear least squares method was used order to determine $B_{0}$ with a fixed $B_{0}^{\prime }$ = 5.29. The fit converged with an R-squared value of 0.9968 with $B_{0}$ = 167.6 ± 5.3 GPa. The third-order Birch-Murnaghan equation of state fit for the radiography data is shown as a solid curve in Figure 2. The equation of state derived from ultrasonic value of bulk modulus and its pressure derivative [8] is also shown in Figure 2. 

The experimental data on equation of state of pure α-Iron presented in Figure 2 for three different methods is summarized in Table 1. The consistency of fitted equation of state (EOS) parameters for three different methods is evident in Table 1. The first derivative of the bulk modulus $B_{0}^{\prime }$ was fixed at ultrasonically measured value of 5.29 for the X-ray radiography and X-ray diffraction EOS fits to obtain a quantitative comparison of the bulk modulus $B_{0}$ obtained from these methods to the ultrasonic measured value. The coefficient of determination for the X-ray radiography and X-ray diffraction data EOS fit is $r^{2}$ = 0.997 and $r^{2}$ = 0.9978, respectively.

The percent difference between the reference bulk modulus from the ultrasonic data and the bulk modulus obtained from radiography measurements is 0.72%. The percent difference between the bulk modulus from the ultrasonic data and the bulk modulus obtained from X-ray diffraction measurements is 1.21%. The percent difference between the bulk modulus obtained from radiography measurements and the bulk modulus obtained from EDXD measurements is 1.93%. Overall, the percent difference between these methods is small, indicating a good agreement between the methods and validating the radiography technique as a method to obtain the equation of state of a solid sample.

### 2.1. Reprocessed Borosilicate Glass Sample

**Figure 3:**
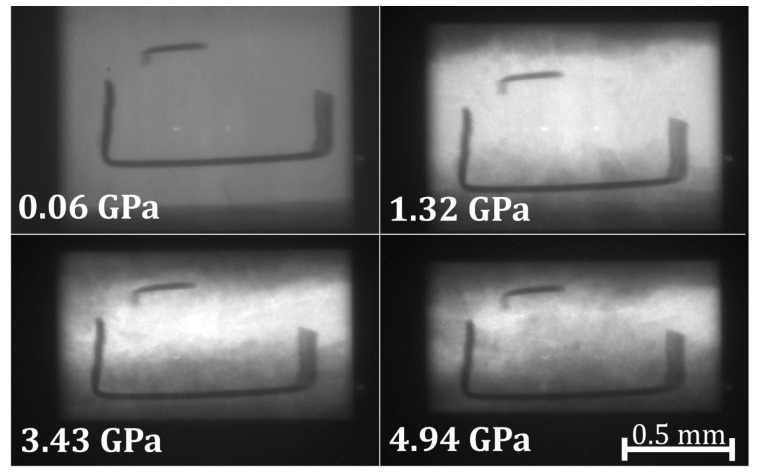
X-ray radiography images of the reprocessed borosilicate glass sample to 4.94 GPa.

The radiography images of the reprocessed borosilicate glass sample at increasing pressure steps are seen in Figure 3. The heights and widths were normalized by the initial height $H_{0}$ and $W_{0}$, respectively, and $\frac{H}{H_{0}}$ and $\frac{W}{W_{0}}$ decrease with an increasing pressure. 

**Figure 4:**
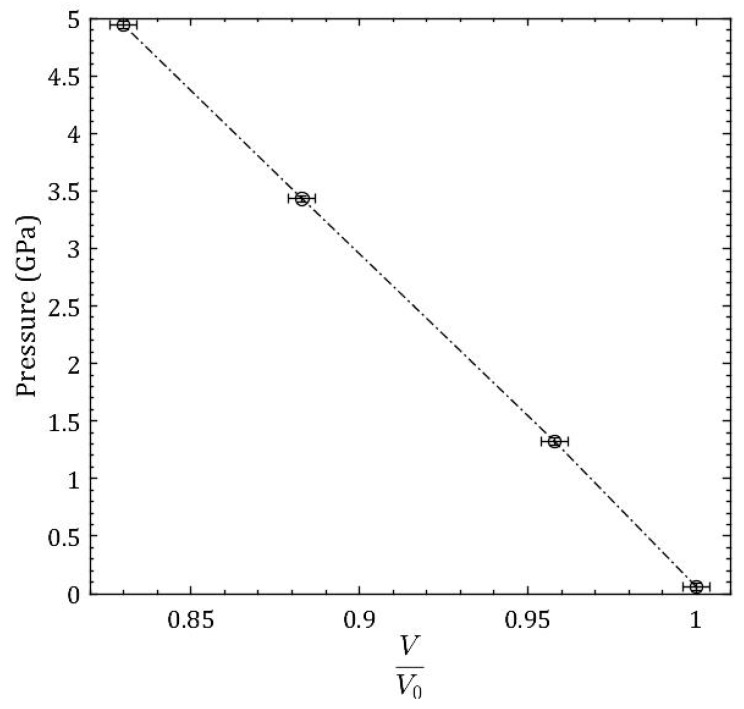
Pressure-Volume (P-V) data or equation of state of reprocessed borosilicate glass obtained by X-ray radiography method. The bulk modulus for the reprocessed borosilicate glass is obtained from the initial compression to 1 GPa.

The volumes were calculated as $V=\pi {\left(\frac{W}{2}\right)}^{2}H$ with the raw height and width values in pixels, and then normalized by the initial volume $V_{0}$ such that $\frac{V}{V_{0}}$ = 1 (Figure 4). The bulk modulus was calculated as $B_{0}=-V{\left(\frac{dP}{dV}\right)}_{0}$ using the low pressure experimental points below 1 GPa, which remain in the elastic region of compression. The bulk modulus obtained is $B_{0}$ = 30.34 GPa.

The sample of commercial borosilicate glass has Young’s modulus *E* = 63.1 GPa and Poisson’s ratio *ν* = 0.18 [9]. Using the equation relating Bulk Modulus *B*_0_ = *E*/[3(1 − 2*ν*)] to the Young’s modulus and Poisson’s ratio, we obtain $B_{0}$ = 32.9 GPa. Using the direct volume measurement from radiography images technique, the bulk modulus obtained for the reprocessed borosilicate glass sample is $B_{0}$ = 30.34 GPa. This gives an 8.1% difference between the experimentally obtained value in this experiment and the value that was obtained through calculation of elastic constants [9]. 

### 2.2. Density Measurements

**Figure 5:**
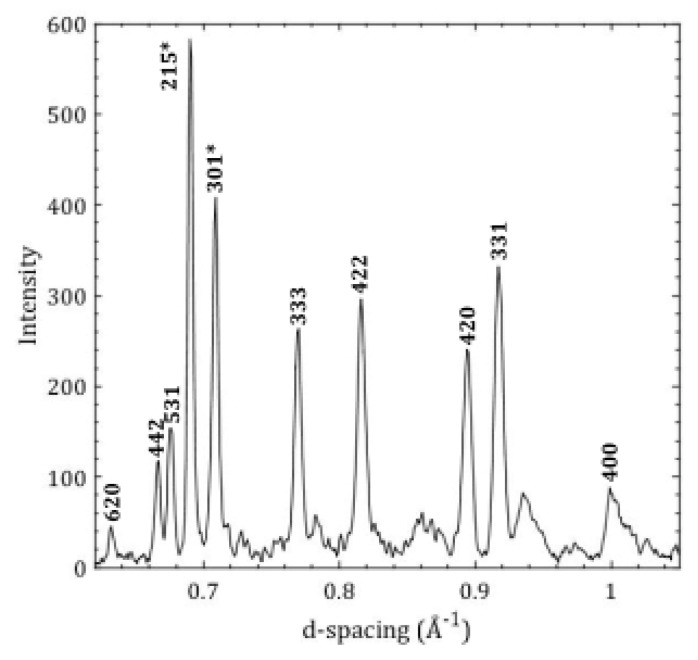
The observed diffraction peaks from the gold pressure marker at 11.4 GPa. The *(hkl)* indices for gold diffraction peaks are indicated and a peak marked ‘*’ is from hexagonal boron nitride sample holder. The measured lattice parameter for gold is *a* = 3.999 Å.

**Figure 6:**
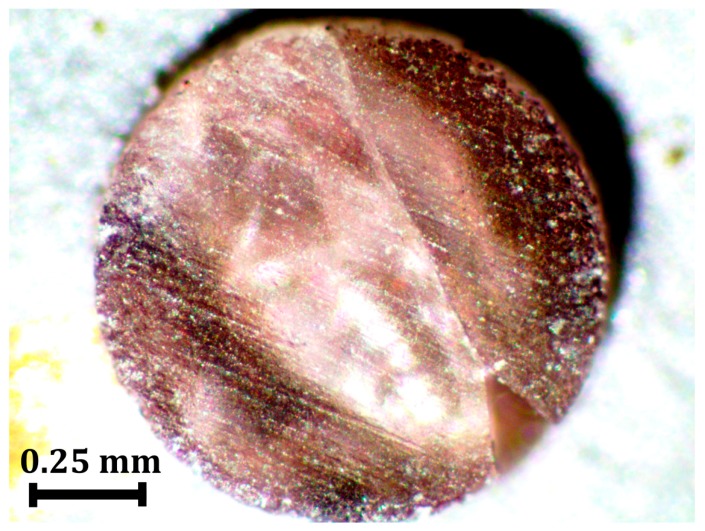
Sample of pressure-treated borosilicate glass after compression to 11.4 GPa. This sample was employed in measurement of pressure induced densification.

Flotation density measurements were conducted on a reprocessed borosilicate glass that was subjected to high pressures in a PE press. The cylindrical borosilicate glass sample was housed in the cell assembly, with a sample diameter of 1.0 mm and a sample height of 1.0 mm and compressed using a PE press and the gold diffraction pattern at the highest pressure is shown in Figure 5. The measured lattice parameter for gold is 3.999 Å for the data shown in Figure 5, and when combined with the gold equation of state [2] yields a pressure of 11.4 GPa. The photograph of the pressure recovered sample from 11.4 GPa is shown in Figure 6.

Lithium metatungstate was the high specific gravity fluid used in density measurements. Deionized water was used to change the density of the fluid throughout the experiment. The sample floated until the final density step, 2.755 gm/cc where the sample was just barely submerged under the surface of the liquid. The flotation density measurements give a final density for the recovered borosilicate glass sample of 2.755 gm/cc. The reprocessed borosilicate glass has an initial density of 2.214 gm/cc, as determined by Archimedes method of the bulk sample. This indicates a 24.4% increase in the initial density for the reprocessed borosilicate glass sample after decompression from 11.4 GPa.

## 3. Discussion

Pressure induced densification and compression in a reprocessed borosilicate glass has been studied using X-ray radiography and X-ray diffraction techniques at a synchrotron source. Borosilicate sample recovered from pressure of 11.4 GPa shows an increase in density of as much as 24%. Direct volume measurements by X-ray radiography combined with pressure measurements by gold pressure marker reveals a bulk modulus of 30.3 GPa, which is in good agreement with value derived from the elastic constants. This methodology of measuring densification and compression can be applied to borosilicate glasses of different compositions. 

## 4. Materials and Methods

The validation of X-ray radiography method was carried on a cylindrical sample of 99.995% pure α-Iron wire from Alfa Aesar (Tewksbury, MA, USA). The α-Fe wire had a diameter of 1.0 mm and a height of 0.5 mm. α-Fe has a body centered cubic structure at ambient condition and retains this structure to 6.3 GPa. The borosilicate glass samples used in this experiment are reprocessed versions of commercially available borosilicate glass [10]. The borosilicate glass was reprocessed by U.S. Army Research Laboratory in Aberdeen Proving Ground, MD to provide base-line data for comparison with glasses of various compositions and to obtain a more optimized glass for use as transparent armor material. The bulk starting glass was broken up and then subjected to reprocessing via cyclical melting and cooling of the same sample in a furnace with the following temperatures and times seen in Table 2. The glass started at 5.25 °C, increased to 1540 °C, and held for 17.0 h, this cycle was repeated on the same glass sample for the amount of time seen in Table 2. The chemical analysis of the borosilicate glass sample, performed via inductively coupled plasma atomic emission spectroscopy, determined a composition of: 2.40% Al_2_O_3_, 12.45% B_2_O_3_, 0.02% BaO, 0.01% Fe_2_O_3_, 0.57% K_2_O, 3.40% Na_2_O, 81.10% SiO_2_, and 0.03% ZrO_2_ (% weight). The cylindrical borosilicate glass sample studied by white-beam radiography had a 1.0 mm diameter and a 0.5 mm height, the borosilicate sample compressed to 11.4 GPa and studied via flotation density measurements had a 1.0 mm diameter and a 1.0 mm height.

### White-Beam X-ray Radiography

**Figure 7:**
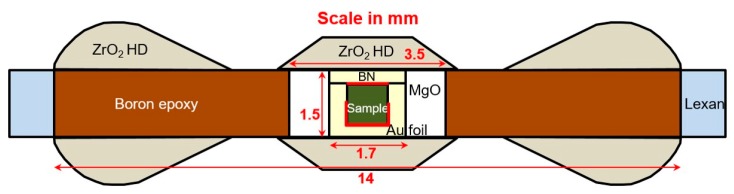
A schematic illustration of the sample assembly utilized in the PE press. This exact assembly was used for the 0.5 mm tall, 1.0 mm diameter cylindrical borosilicate glass sample.

White-beam X-ray radiography studies were conducted at Beamline 16-BM-B, HPCAT (Argonne, IL, USA), The Advanced Photon Source, Argonne National Laboratory, on a sample of α-Fe and a sample of reprocessed borosilicate glass. Both of the samples were quasi-hydrostatically compressed at ambient temperature using a Paris-Edinburgh (PE) cell. The cell assembly, seen in Figure 7, consists of a cylindrical glass sample housed within a hexagonal boron nitride (h−BN) cup with a h−BN cap, which is surrounded by a magnesium oxide (MgO) inner ring and a boron epoxy outer ring, which is all surrounded by supporting outer polycarbonate plastic (Lexan) ring; this setup is sandwiched between zirconium oxide (ZrO_2_) caps, which are shaped to match the PE anvil geometry. 

Gold foil (2 µm thick, shown in red in Figure 7), an important component of this cell assembly, was used as the pressure standard [2] and the marker in the white-beam X-ray radiography direct volume measurement. Two separate pieces of gold foil were used, one on top of the sample and one longer piece that fit underneath of the sample in a ’U’ shape, in order to directly measure the changing sample height and width with increasing pressure. In between each white-beam X-ray radiography measurement an energy-dispersive X-ray diffraction (EDXD) spectrum was taken of the top gold foil at *2θ* = 15.01°, in order to determine pressure. The (220), (311), (222), (400), (331), (420), (422), and (333) Miller indices *(hkl)* were indexed for gold foil (space group $Fm\bar{3}m$ (number 225)) to determine the lattice parameter *a* in order to obtain the volume *V*. This volume was used with bulk modulus $B_{0}$ = 165.8 GPa, first derivative of the bulk modulus $B_{0}^{\prime }$ = 5.14, and the initial unit-cell volume $V_{0}$ = 67.850 A [3] (at ambient conditions) to obtain the sample pressure using the third-order Birch-Murnaghan equation of state [1]. 

## 5. Conclusions

A proof of concept experiment was conducted on a sample of pure α-Fe to check the validity of the use of X-ray radiography to obtain an equation of state for amorphous materials. The bulk modulus that were obtained through a third-order Birch-Murnaghan equation of state fit to experimentally obtained volume data by radiography, X-ray diffraction, and ultrasonic method showed excellent agreement. Direct volume measurements of a reprocessed borosilicate sample were conducted via whitebeam X-ray radiography to a pressure of 4.9 GPa. From the initial compression, we obtain bulk modulus for the borosilicate glass sample to be B_0_ = 30.3 GPa. The percent difference between the bulk modulus of the reprocessed borosilicate sample obtained via whitebeam X-ray radiography and the commercial borosilicate sample is 8.1%. It is important to note that the borosilicate glass that was studied in this current research was a reprocessed version of the commercially available, which could contribute to the 8.1% difference in the bulk modulus. The experimentally obtained density of the recovered borosilicate glass sample compressed to 11.4 GPa was 2.755 gm/cc and showed densification of 24% when compared to starting materials. In conclusion, direct volume measurements via white-beam X-ray radiography prove an effective method to obtain the equation of state of amorphous materials. The method described in this paper can be applied to obtain densification and compression data on a broad cross-section of amorphous materials.

## Figures and Tables

**Table 1:** Measured equation of state (EOS) parameters for α–Iron by three different methods.

EOS Parameters	X-ray Radiography	X-ray Diffraction	Ultrasonic [8]
$B_{0}$ **(GPa)**	167.6	164.4	166.4
$B_{0}^{\prime }$	5.29 (fixed)	5.29 (fixed)	5.29

**Table 2:** Reprocessing Furnace Cycle for borosilicate glass.

Heating Rate (°C Per Minute)	Ending Temperature (°C)	Time * (h)
5.25	1540	17.0
0.9	1090	4.5
0.7	935	3.5
0.5	820	3.5
0.3	590	14.0
0.2	535	8.5
0.2	450	5.5
0.2	25	End

* Time held at ending temperature (°C).

## References

[1] Birch F. (1947). Finite elastic strain of cubic crystals. Phys. Rev..

[2] Fei Y., Li J., Hirose K., Minarik W.G., Van Orman J.A., Sanloup C., Westrenen W., van Komabayashi T., Funakoshi K. (2004). A critical evaluation of pressure scales at high temperatures by in situ X-ray diffraction measurements. Phys. Earth Planet. Inter..

[3] Amin S.A., Rissi E.N., McKiernan K., Yarger J.L. (2012). Determining the equation of state of amorphous solids at high pressure using optical microscopy. Rev. Sci. Instrum..

[4] Liu H., Wang L., Xiao X., De Carlo F., Feng J., Mao H., Hemley R.H. (2008). Anomalous high-pressure behavior of amorphous selenium from synchrotron X-ray diffraction and microtomography. Proc. Natl. Acad. Sci. USA.

[5] Kono Y., Yamada A., Wang Y., Yu T., Inoue T. (2011). Combined ultrasonic elastic wave velocity and microtomography measurements at high pressures. Rev. Sci. Instrum..

[6] Li L.L., Weidner D., Raterron P., Chen J.H., Vaughan M., Me S.H., Durham B. (2006). Deformation of olivine at mantle pressure using the D-DIA. Eur. J. Mineral..

[7] Ham K.J., Vohra Y.K., Kono Y., Wereszczak A., Patel P. (2017). White-beam X-ray diffraction and radiography studies on high-boron-containing borosilicate glass at high pressures. High Press. Res..

[8] Guinan M.W., Beshers D.N. (1968). Pressure derivatives of the elastic constants of α-iron to 10 kbs. J. Phys. Chem. Solids.

[9] Wereszczak A.A., Anderson C.E. (2015). Borofloat and starphire float glasses: A comparison. Int. J. Appl. Glass Sci..

[10] SCHOTT Technical Glass Solutions GmbH (2018). Schott Borofloat R 33.

